# Mortality among Workers Exposed to Polychlorinated Biphenyls (PCBs) in an Electrical Capacitor Manufacturing Plant in Indiana: An Update

**DOI:** 10.1289/ehp.8253

**Published:** 2005-09-01

**Authors:** Avima M. Ruder, Misty J. Hein, Nancy Nilsen, Martha A. Waters, Patricia Laber, Karen Davis-King, Mary M. Prince, Elizabeth Whelan

**Affiliations:** National Institute for Occupational Safety and Health, Cincinnati, Ohio, USA

**Keywords:** cancer, cohort study, exposure assessment, occupational exposure, polychlorinated biphenyls

## Abstract

An Indiana capacitor-manufacturing cohort (*n* = 3,569) was exposed to polychlorinated biphenyls (PCBs) from 1957 to 1977. The original study of mortality through 1984 found excess melanoma and brain cancer; other studies of PCB-exposed individuals have found excess non-Hodgkin lymphoma and rectal, liver, biliary tract, and gallbladder cancer. Mortality was updated through 1998. Analyses have included standardized mortality ratios (SMRs) and 95% confidence intervals (CIs) using rates for Indiana and the United States, standardized rate ratios (SRRs), and Poisson regression rate ratios (RRs). Estimated cumulative exposure calculations used a new job–exposure matrix. Mortality overall was reduced (547 deaths; SMR, 0.81; 95% CI, 0.7–0.9). Non-Hodgkin lymphoma mortality was elevated (9 deaths; SMR, 1.23; 95% CI, 0.6–2.3). Melanoma remained in excess (9 deaths; SMR, 2.43; 95% CI, 1.1–4.6), especially in the lowest tertile of estimated cumulative exposure (5 deaths; SMR, 3.72; 95% CI, 1.2–8.7). Seven of the 12 brain cancer deaths (SMR, 1.91; 95% CI, 1.0–3.3) occurred after the original study. Brain cancer mortality increased with exposure (in the highest tertile, 5 deaths; SMR, 2.71; 95% CI, 0.9–6.3); the SRR dose–response trend was significant (*p* = 0.016). Among those working ≥90 days, both melanoma (8 deaths; SMR, 2.66; 95% CI, 1.1–5.2) and brain cancer (11 deaths; SMR, 2.12; 95% CI, 1.1–3.8) were elevated, especially for women: melanoma, 3 deaths (SMR, 5.99; 95% CI, 1.2–17.5); brain cancer, 3 deaths (SMR, 2.87; 95% CI, 0.6–8.4). These findings of excess melanoma and brain cancer mortality confirm results of the original study. Melanoma mortality was not associated with estimated cumulative exposure. Brain cancer mortality did not demonstrate a clear dose–response relationship with estimated cumulative exposure.

Polychlorinated biphenyls (PCBs) are synthetic chemicals that were produced commercially in the United States from 1929 to 1977 and used widely in the electrical industry because of their high stability, dielectric properties, and resistance to oxidation (U.S. EPA and Environment Canada 2004). They were also used in plasticizers, adhesives, and hydraulic fluids (Silberhorn et al. 1990; Smith and Brown 1987). PCBs have long half-lives, correlated with the degree of chlorination, and persist in humans and in the environment (Brown and Lawton 2001; Hansen 1998). Increasing concern in the 1970s about potential health and environmental risks led to a 1977 ban on PCB production and distribution in the United States.

The International Agency for Research on Cancer (IARC) classified PCBs as probable human carcinogens (2A) with sufficient evidence of carcinogenicity in animals but limited evidence from human studies (IARC 1987). The U.S. Environmental Protection Agency (EPA) also classified PCBs as probable human carcinogens [Integrated Risk Information System (IRIS) 1996]. The National Toxicology Program (NTP) has classified several PCB mixtures as “reasonably anticipated to be human carcinogens” since 1981 (NTP 2005).

The human carcinogenicity of PCBs remains an important issue. Almost 30 years after production was banned, PCBs are still a potential occupational exposure. According to the U.S. EPA and Environment Canada (2004), at least 44% (87,000) of PCB transformers and 10% (143,000) of PCB capacitors were disposed of between 1994 and 2000. However, as many as 113,000 PCB transformers and 1.33 million PCB capacitors may still be in use. Those who repair and maintain capacitors and transformers containing PCBs and those in the reclamation industry responsible for disassembly of PCB-containing capacitors and transformers have the highest potential for exposure.

This update of a cohort mortality study among workers exposed to PCBs in an electrical capacitor manufacturing plant in Indiana was undertaken because the carcinogenicity of PCBs in humans is unresolved, and because on initial follow-up, both potential latency and statistical power were limited. The primary purpose was to investigate further the increased risks for brain cancer and malignant melanoma originally observed in the cohort followed through 1984 (Sinks et al. 1992). Other *a priori* hypotheses were that PCB exposure would affect all-cause mortality, all cancer mortality, and, specifically, rectal, liver, biliary tract, and gallbladder cancer and non-Hodgkin lymphoma, for which other studies indicated increased risks (Brown 1987; Brown and Jones 1981; Rothman et al. 1997). We updated mortality through 1998, adding 14 years of follow-up.

## Materials and Methods

Capacitors were manufactured at an Indiana facility, using PCBs as a dielectric fluid from fall 1957 until spring 1977, when PCBs were replaced with isopropyl biphenyl (Jones 1977). Two dielectric fluid formulations were used, Aroclor 1242 through 1971 and Aroclor 1016 from 1971 to 1977 (Jones 1977). In both formulations, dichlorobiphenyls to tetrachlorobiphenyls predominated, but Aroclor 1242 contained 5.5% pentachlorinated and hexachlorinated biphenyls versus 0.4% pentachlorinated biphenyls for Aroclor 1016 (Albro and Parker 1979; Hutzinger et al. 1985). Aroclor composition could vary from batch to batch (de Voogt and Brinkman 1989; Kimbrough 1995).

Capacitor production began by winding foil and film into bales in a dust-free room with minimal exposure to PCBs, placing bales in metal capacitor boxes, and welding boxes shut. Capacitors were impregnated with dielectric fluid in a heated vacuum chamber. Large capacitors requiring gallons of dielectric fluid were filled manually through ports on the top (reportedly resulting in spillage and extensive dermal contact with dielectric fluid). The ports of the filled, warm, wet capacitors were soldered shut, dielectric fluid was washed off the outside, and capacitors were sent to quality control for testing. No regular industrial hygiene monitoring was done at the facility. All operations were under one roof with partitions between operations. The administrative offices and a few specific processes were isolated by walls (Jones 1977).

In spring 1977, the National Institute for Occupational Safety and Health (NIOSH) collected 9 skin smear samples, 16 area samples, and 40 personal air samples to evaluate exposures to PCBs and other chemicals. Low levels of xylene (mean, 1.8 ppm) and toluene (mean, 2.7 ppm) were found for painters, and appreciable levels of 1,1,1-trichloroethane (7–339 ppm) were found in the degreaser area and trichloroethylene (62–290 ppm) in the plating and welding areas (Jones 1977).

Exposure assessment for the original mortality study (Sinks et al. 1992) was based on duration of employment in jobs judged to have high direct PCB exposure (impregnating, sealing, and testing capacitors), based on personal and area air sampling. About 10% of the work force was estimated to have had such exposure (Sinks et al. 1992).

Exposure assessment for this update was based on a newly created semiquantitative job–exposure matrix (JEM) (Nilsen et al. 2004). All unique jobs (*n* = 884) were categorized based on PCB exposure intensity and frequency, qualitatively ranked for both inhalation and dermal exposure. For inhalation exposure intensity, air concentration data permitted assignment of exposure units (parts per million), but for dermal exposure intensity, the lack of historical dermal exposure measurements resulted in a unitless measure of exposure. For each job category, the product of intensity and frequency (fraction of day exposed) was calculated. The inhalation and dermal JEMs were modified for an earlier and a later era (the former with estimated 20% higher exposure). Because dermal exposures account for a significant proportion of total PCB exposure (Fischbein et al. 1982), a combination JEM averaging inhalation and dermal (1:1) scores was used to estimate cumulative PCB exposure. Cumulative exposure was expressed in unit-days of exposure (but the “unit” was not defined).

The cohort includes 3,569 of the 3,643 workers ever employed at the facility (74 were ineligible). For the original study (Sinks et al. 1992), vital status was ascertained through 1984. For the update, we submitted names of cohort members to the National Death Index (NDI 2005) for determination of vital status through 31 December 1998, and obtained death certificates or NDI-Plus causes of death (CODs). NDI-Plus searches retrieve COD codes as well as date of death. Death certificate data were coded by a nosologist. Because the NDI does not include deaths before 1979, any worker lost to follow-up before 1979 was classified “vital status unknown” and considered alive until the date last observed (usually the date last employed). Death was coded to the revision of the *International Classification of Diseases, 9th Revision* [ICD-9; World Health Organization (WHO) 1979] in effect at the time of death. This study was approved by the NIOSH Human Subjects Review Board. As a records study, it was exempted from informed consent requirements.

### Statistical analysis.

The standardized mortality ratio (SMR) is the ratio of observed to expected deaths. Sex/race/age/calendar period reference rate files based on mortality in the Indiana and U.S. populations include 99 CODs, each encompassing a number of ICD codes, and cover the period beginning in 1960. State rates control for local conditions that may have no association with occupational exposures. In addition to the gradient of disease with latitude seen for infectious diseases (Guernier et al. 2004) and some cancers (Nomura and Kolonel 1991; Schwartz 1992), regional differences can affect other CODs (Mansfield et al. 1999; Pickle et al. 1997). We present Indiana-based SMRs, except as noted.

Our analyses used the NIOSH PC Life Table Analysis System (Cassinelli et al. 1997; NIOSH 2001; Steenland et al. 1990, 1998; Waxweiler et al. 1983). The statistical significance of the SMR was determined by a two-tailed test based on the Poisson distribution. The program calculated the 95% confidence interval (CI) for each SMR estimate.

Race- and sex-specific person-years at risk (PYAR) were accumulated for each eligible worker across 5-year age and calendar year intervals, beginning on 1 January 1960 or the qualified date of first exposure, whichever was later, and ending with the date of death, the date last known alive, or 31 December 1998, the study end date. Cohort members known to be alive after 1 January 1979 and not identified as deceased were assumed to be alive on 31 December 1998. Latency began at the date of first exposure and ended with the date of death, the date last known alive, or on 31 December 1998. For analyses we used the Indiana and U.S. 99-COD rate files. Using a multiple-COD analysis (MCOD) and U.S. rates (Steenland et al. 1992), we investigated possible excesses in nonmalignant chronic diseases. A separate analysis was restricted to individuals who worked at least 90 days (*n* = 2,789) *a*) to facilitate comparison with analyses of New York and Massachusetts cohorts of capacitor manufacturing workers exposed to PCBs (Brown 1987; Brown and Jones 1981; Kimbrough et al. 1999, 2003) and *b*) because there appear to be lifestyle and mortality differences between short-term and long-term workers (Kolstad and Olsen 1999).

We calculated estimated cumulative PCB exposure for each worker, based on job titles, job codes, and era(s) of employment. Cumulative exposure ranged from 10 to 1,218,590 unit-days of PCB exposure (median, 16,860 unit-days) (Nilsen et al. 2004). Cut-points at 11,000 and 90,000 unit-days of exposure defined tertiles with approximately equal numbers of deaths. Standardized rates were calculated for each cumulative exposure tertile using the sum of all PYAR for each sex/race/age/calendar time stratum as the weight for the specific stratum. Standardized rate ratios (SRRs) were calculated for each higher exposure tertile relative to the lowest tertile. Based on a Taylor series approximation of the variance, 95% CIs were calculated and a test for a linear trend was performed based on a weighted regression of the standardized rates (Rothman 1989). We used multivariate Poisson regression modeling and SAS 9 software (SAS Institute 2004) to adjust for sex, age, calendar year, and latency, and to calculate rate ratios (RRs) for higher exposure tertiles relative to the lowest tertile. We repeated the analysis excluding 117 workers with potential exposure to solvents (xylene, toluene, 1,1,1-trichloroethane, and trichloroethylene).

We used original department and operation codes from plant records and a map of exposure zones developed for the original study (Sinks et al. 1992) to re-create exposure zone assignments. The mortality analysis was repeated using exposure zones.

## Results

Table 1 shows the cohort stratified by race, sex, and vital status. About one-third of the cohort (1,176 workers, 33%) worked between 1 day and 6 months, one-third (1,133 workers, 32%) 6 months to 3 years, and one-third (1,260 workers, 35%) > 3 years. Nearly all (97%) in the highest tertile of estimated cumulative exposure worked > 3 years, and nearly all (93%) in the lowest exposure tertile worked < 3 years.

About 6% of men’s work-years and 1.2% of women’s were in the highest exposure jobs (salvage and repair; fill, solder, impregnate; leak tester). Men had a mean cumulative exposure of 82,503 unit-days; the mean for women was 47,824 unit-days; 23% of men’s and 16% of women’s PYAR fell into the highest exposure tertile. For 221 workers who provided blood specimens in 1977 (Smith et al. 1982), estimated cumulative exposure and serum PCB level were significantly correlated (Spearman correlation, *r* = 0.37, *p* < 0.0001); serum PCB level and duration of exposure were not well correlated (Spearman correlation, *r* = 0.10, *p* = 0.15).

Observed deaths, corresponding SMRs using Indiana rates, and the SMR CIs are presented in Table 2 for the three exposure tertiles and overall. Mortality overall was reduced (547 deaths; SMR, 0.81; 95% CI, 0.7–0.9). In race- and sex-specific analyses (not shown), the overall statistics for white males and females were 453 deaths (SMR, 0.82; 95% CI, 0.7–0.9) and 84 deaths (SMR, 0.74; 95% CI, 0.6–0.9), respectively. Two deaths occurred among 19 nonwhite female employees and eight among 11 nonwhite male employees.

No excess deaths due to malignant neoplasms overall were observed (171 deaths; SMR, 0.90; 95% CI, 0.8–1.0). In the MCOD analysis, 268 deaths had cancer as the underlying or contributing cause (MCOD U.S. SMR, 1.00; 95% CI, 0.9–1.1).

Among the *a priori* cancers of interest, melanoma was in statistically significant excess (9 deaths; SMR, 2.43; 95% CI, 1.1–4.6). In the original analysis (Sinks et al. 1992), there were 8 skin cancer deaths (all melanomas) (U.S. SMR, 4.1; 95% CI, 1.8–8.0). Seven brain cancer deaths occurred after the previous report (present update: 12 deaths; SMR, 1.91; 95% CI, 1.0–3.3; original study: 5 deaths, U.S. SMR, 1.8; 95% CI, 0.6–4.2). The 12 brain cancers included 8 gliomas and 4 carcinomas. Review of the death certificates indicated that 2 of the carcinomas (both in men) could have been metastases. In a sensitivity analysis to determine risk omitting those 2 deaths, brain cancer SMR decreased (10 deaths; SMR, 1.59; 95% CI, 0.8–2.9).

Non-Hodgkin lymphoma mortality, not reported separately in the original study (Sinks et al. 1992), was increased but not statistically significant (9 deaths; SMR, 1.23; 95% CI, 0.6–2.3). No other subcategory of hematopoietic cancers (Hodgkin disease, leukemia and aleukemia, or myeloma) showed excess deaths (results not shown). Other cancers of *a priori* interest (rectal and biliary passages, liver, and gallbladder) were not in excess.

As is typical of a working population, the cohort overall had no statistically significant increased SMRs for diseases other than cancer and generally decreased SMRs for heart diseases, especially ischemic heart disease (149 deaths; SMR, 0.84; 95% CI, 0.7–1.0). Cardiomyopathy mortality was elevated (13 deaths; SMR, 1.67; 95% CI, 0.9–2.9), with a significant excess in the lowest exposure tertile (7 deaths; SMR, 2.79; 95% CI, 1.1–5.7). There were decreased risks for deaths from other circulatory system diseases (27 deaths; SMR, 0.56; 95% CI, 0.4–0.8); digestive system diseases (14 deaths; SMR, 0.51; 95% CI, 0.3–0.9), including cirrhosis of the liver (6 deaths; SMR, 0.43; 95% CI, 0.2–0.9); and homicide (3 deaths; SMR, 0.46; 95% CI, 0.1–1.3).

In analyses restricted to 2,789 employees who worked at least 90 days, mortality overall was reduced (445 deaths; SMR, 0.79; 95% CI, 0.7–0.9), as was cancer overall (136 deaths; SMR, 0.85; 95% CI, 0.7–1.0). Both melanoma (8 deaths; SMR, 2.66; 95% CI, 1.1–5.2) and brain cancer (11 deaths; SMR, 2.12; 95% CI, 1.1–3.8) were in excess, especially among women: 3 melanoma deaths (SMR, 5.99; 95% CI, 1.2–17.5) and 3 brain cancer deaths (SMR, 2.87; 95% CI, 0.6–8.4). Eliminating 1 male brain cancer death that could have been a metastasis would change the overall SMR to 1.93 (95% CI, 0.9–3.6) and the SMR among men to 1.69 (95% CI, 0.7–3.5). The non-Hodgkin lymphoma mortality increase was not statistically significant (8 deaths; SMR, 1.31; 95% CI, 0.6–2.6), but the rate was higher among women (3 deaths; SMR, 2.42; 95% CI, 0.5–7.1). As in the cohort overall, mortality from heart disease, digestive system disease, and homicide was reduced (results not shown). Cardiomyopathy mortality remained elevated (10 deaths; SMR, 1.55; 95% CI, 0.7–2.8) and occurred exclusively in men (SMR, 1.82; 95% CI, 0.9–3.3).

Table 2 provides Indiana-based SMRs for the three exposure tertiles for all CODs. For both overall mortality and all cancer, there was no significant trend with increasing estimated cumulative exposure (Table 3). Table 3 presents SRRs and RRs (adjusted for sex, age, calendar year, and latency) for melanoma and brain cancer. Melanoma was in excess in the lowest tertile (5 deaths; SMR, 3.72; 95% CI, 1.2–8.7), but the trend test was not significant. Brain cancer mortality increased with exposure (5 deaths in the highest tertile; SMR, 2.71; 95% CI, 0.9–6.3); 3 of the 5 deaths were among women. SMRs, SRRs, and RRs increased with increasing exposure, but only the SRR dose–response trend was statistically significant (*p* = 0.016). In the sensitivity analysis, when we excluded 2 brain cancer deaths that could have been metastases, results by tertile changed [lowest tertile: 2 deaths; SMR, 0.92 (95% CI, 0.1–3.3); SRR 1.0; middle tertile: 4 deaths; SMR, 1.79 (95% CI, 0.5–4.6); SRR 1.64 (95% CI, 0.3–9.0); highest tertile: 4 deaths; SMR, 2.17 (95% CI, 0.6–5.6); SRR 1.96 (95% CI, 0.4–11.1)]. The SRR dose–response trend remained statistically significant (*p* = 0.01).

We reran the analysis excluding those (*n* = 117) with potential solvent exposure. No melanoma or brain cancer deaths occurred among the solvent-exposed workers. Results for all deaths and all cancer deaths did not change significantly (data not shown).

We repeated the estimated cumulative exposure analysis using the exposure zones developed for the original study. Results (data not shown) were similar to those using the JEM: brain cancer was associated with higher levels of exposure, but no dose–response relationship between estimated cumulative PCB exposure and melanoma was found. Analysis of the 153 deaths among the 1,139 workers who ever worked in the highest exposure zone identified in the original study (Sinks et al. 1992) demonstrated no increase in risk with time worked for all deaths or cancer deaths (data not shown). Comparing the original study exposure model with the two JEMs gave Spearman correlations of *r* = 0.6 (*p* < 0.0001) for the inhalation JEM and *r* = 0.4 (*p* < 0.0001) for the dermal JEM.

For cancer overall, stratifying by latency (time from first exposure to death) did not affect mortality [< 10 years: 11 deaths; SMR 0.80 (95% CI, 0.4–1.4); 10 to < 20 years: 37 deaths, SMR 1.03 (95% CI, 0.7–1.4); ≥20 years: 122 deaths, SMR 0.88 (95% CI, 0.7–1.0)]. Brain cancer mortality was elevated in each latency period [< 10 years: 2 deaths, SMR 2.44 (95% CI, 0.3–8.8); 10 to < 20 years: 4 deaths, SMR 2.63 (95% CI, 0.7–6.7); ≥ 20 years: 6 deaths, SMR 1.53 (95% CI, 0.6–3.3)]. Melanoma mortality was elevated among workers with shorter but not longer latency [< 10 years: 2 deaths, SMR 4.71 (95% CI, 0.6–17.0); 10 to < 20 years: 5 deaths, SMR 5.03 (95% CI, 1.6–11.7); ≥ 20 years: 2 deaths, SMR 0.88 (95% CI, 0.1–3.2)].

## Discussion

Studies of mortality in cohorts occupationally exposed to PCBs present inconsistent findings. Nine cohorts of electrical capacitor and transformer manufacturers have been studied in, to date, 17 reports in the literature or in unpublished documents [see Supplemental Material (http://ehp.niehs.nih.gov/docs/2005/8253/supplement.pdf)]. In some cases SMRs were elevated for one sex but not the other. Excess deaths from particular cancers or other diseases have been reported, but there has been little consistency from cohort to cohort, or even within cohorts across studies.

Brown (1987) found excess liver cancer among the high-exposed group (1,607 workers) within a Massachusetts capacitor manufacturing cohort (4 deaths; SMR, 3.3; 95% CI, 0.9–9.3), with all four deaths occurring in women (SMR, 4.4; 95% CI, 1.2–12.3). Taylor et al. (1988) expanded the New York cohort to all those working 90 days or more (6,292 workers) and found a slight excess of digestive system cancer (44 deaths; SMR, 1.3; 95% CI, 1.0–1.8), whereas Kimbrough et al. (1999, 2003), studying a reexpanded New York cohort (*n* = 7,075) reported no cancer SMR excesses.

Three studies of the same Italian factory (Bertazzi et al. 1982, 1987; Tironi et al. 1996) reported elevated SMRs for lymphatic and hematopoietic cancers, and digestive system cancers in men. Swedish male capacitor manufacturing workers (Gustavsson et al. 1986; Gustavsson and Hogstedt 1997) had no increased mortality risk, whereas Canadian transformer manufacturing workers had excess pancreatic cancer, especially in the transformer assembly department (4 deaths; SMR, 9.8; 95% CI, 2.6–25) (Yassi et al. 1994). Transformer manufacturing workers in Massachusetts exposed to PCBs had an odds ratio of 3.3 (95% CI 1.1–9.3) for lymphoma, compared with co-workers not PCB-exposed (Greenland et al. 1994). In an Illinois capacitor manufacturing facility, Mallin et al. (2004) found excess gastrointestinal cancer, especially among those working 5 or more years between 1952 and 1977 [men, 3 stomach cancer deaths (SMR, 3.09; 95% CI, 0.6–9.0); women, 9 intestinal cancer deaths (SMR, 2.25; 95% CI, 1.0–4.3) and 4 liver cancer deaths (SMR, 5.57; 95% CI, 1.5–14.3)]. The original report on our cohort found three skin cancer (all melanoma) deaths (SMR, 7.0; 95% CI, 1.4–23) and three brain cancer deaths (SMR, 4.8; 95% CI, 1.0–16) among those employed at least 10 years (Sinks et al. 1992).

Some of these cohort studies are uninformative due to small sample size, insufficient latency, or problems in study design. Differences in findings between cohorts could be due to differences in materials or work practices; each plant potentially had a unique pattern of exposures. Although air concentrations are expressed in standard units that permit comparisons across plants, dermal exposure measurements are not, and dermal exposure is a significant route for PCBs (Lees et al. 1987; Safe 1984; Smith et al. 1982; Wolff et al. 1982). Different results in studies of the same cohort could be due to variations in study eligibility criteria, in choice of comparison groups, or in how results were presented.

Several studies have reported significantly higher serum or adipose tissue levels of PCBs in cancer cases than in controls (Aronson et al. 2000; Charlier et al. 2004; Howsam et al. 2004; Rothman et al. 1997). Others have seen no association of serum PCB and cancer risk (Dorgan et al. 1999; Gammon et al. 2002; Rusiecki et al. 2004; Ward et al. 2000). It should be noted that in all these studies, whether blood was collected prospectively in the 1970s when PCB use was widespread or retrospectively 20–30 years later when mean serum levels had decreased by 80–90% (Schecter et al. 2005), serum levels among the environmentally exposed would be much lower than among the occupationally exposed. For example, archived 1974 serum samples of Maryland residents had mean levels of 7.56 and 6.55 ng PCBs/mL for non-Hodgkin lymphoma cases and controls, respectively (Rothman et al. 1997), whereas workers from the Indiana plant had mean 1977 levels of 546 and 111 ng PCBs/mL serum for the most and least exposed workers, respectively (Smith et al. 1982).

As in many cohort mortality studies, limited data were available to construct the JEM: individual work histories, detailed job descriptions for hourly jobs, and 56 measurements collected at the plant in 1977. The JEM used proximity to the ovens, as did the zone classification used in the original study (Sinks et al. 1992), but also incorporated job descriptions, plant layouts, workers’ mobility, exposure intensity and frequency, inhalation and dermal exposure to PCBs, and exposure to other chemicals. Analyses replicating the exposure zones from the original study yielded results similar to those using the JEM (brain cancer was associated with higher levels of exposure; there was no dose–response relationship between estimated cumulative PCB exposure and melanoma). Mortality did not increase with time worked in the highest exposure zone. The zone exposure model and inhalation and dermal JEMs were significantly correlated. An independent measure of cumulative exposure is body burden. Serum collected from 221 Indiana workers in 1977 was analyzed for PCBs (Smith et al. 1982). Cumulative exposure estimated with the JEM and serum PCB levels were significantly correlated.

Our findings confirm those of the original study (Sinks et al. 1992) of excess melanoma and brain cancer mortality. Although no other study of capacitor manufacturing workers found elevated SMRs for these sites, a study of transformer manufacturing observed an elevated standardized incidence ratio (SIR) for brain cancer among males ever in PCB-exposed jobs (4 diagnoses; SIR, 4.4; 95% CI, 1.2–12) (Liss 1989). Excess melanoma was reported (Bahn et al. 1976) for PCB-exposed employees of a New Jersey petrochemical plant (2 cases; SIR, 50; 95% CI 5.6–217). Nine employees of Norwegian hydroelectric power plants ever exposed to PCBs had melanoma (SIR, 1.8; 95% CI, 0.8–3.6), with risk concentrated among those who also had > 15 μT years of magnetic field exposure versus fewer years (9 cases; SIR, 2.7; 95% CI, 1.2–5.2) or > 30 exposure-years to electrical discharges versus fewer years (7 cases; SIR, 2.8; 95% CI, 1.1–6.0) (Tynes et al. 1994). Loomis et al. (1997) found excess melanoma mortality in a large cohort of electric utility workers, with increasing risk for increasing cumulative exposure, as well as increased brain cancer risk among workers in the two intermediate (but not the highest) quartiles of exposure.

We found higher SMRs among women for both melanoma and brain cancer. Men, however, generally had jobs with higher exposure as well as higher cumulative PCB exposure. Some interaction between estrogenic PCBs and hormones may contribute to the higher risk for women (Gore et al. 2002; Soontornchat et al. 1994). Because women have a higher percentage of adipose tissue, PCBs may be stored in their bodies longer (Brown 1994).

Our sensitivity analysis of the brain cancer deaths (8 gliomas and 4 carcinomas), excluding 2 carcinomas that could have been metastasis, still found the highest SMRs and SRRs among workers in the highest tertile of estimated cumulative exposure. An analysis excluding 117 workers with potential solvent exposure at the plant did not affect melanoma or brain cancer mortality. Stratifying by latency (time from first exposure to death) did not affect mortality for cancer overall and for brain cancer. Melanoma mortality was elevated among workers with shorter but not longer latency.

As in most cohort studies, we had no information on risk factors such as family history or genetic susceptibility; lifestyle choices, such as sun exposure, that could affect mortality; or on previous or subsequent employment. This last limitation is significant for the two-thirds of the cohort who worked < 3 years in the plant. (It should be noted that 97% of workers in the highest tertile, with the highest brain cancer risk, worked ≥3 years.)

Increased mortality among short-term workers has been reported, particularly for CODs associated with disorders that might affect employment or with an unhealthy lifestyle. Kolstad and Olsen (1999) found that shorter durations of employment were associated with more preemployment hospitalizations for alcohol use, accidents, and the effects of violence. We have no lifestyle or hospitalization information for our cohort. However, when short-term workers were excluded from the analysis, the already low SMRs for cirrhosis of the liver and homicide (Table 2) dropped even farther, whereas the SMRs for melanoma and brain cancer increased.

In conclusion, we found evidence of an association between employment at this plant and melanoma and brain cancer mortality. We used a JEM that incorporated both inhalation and dermal exposure potentials to estimate cumulative exposure. However, melanoma mortality was not associated with estimated cumulative PCB exposure, and for brain cancer, the association between mortality and estimated PCB cumulative exposure did not demonstrate a clear dose–response relationship.

The cancer incidence study we are conducting on this cohort (and the New York and Massachusetts NIOSH cohorts) may provide some additional insight.

## Supplementary Material

Supplementary Material

## Figures and Tables

**Table 1:** NIOSH Indiana capacitor cohort as of 31 December 1998.

Characteristic	No., %		
Total workers	3,643		
Excluded from analysis^a^	74		
Race, sex, and vital status			
No. (no. of deaths, % dead in stratum^b^)			
White females	833 (84, 10%)		
Nonwhite females	19 (2, 11%)		
White males	2,706 (453, 17%)		
Nonwhite males	11 (8, 73%)		
Total analyzed	3,569 (547, 15%)		
Age at first employment (years)			
Median	24		
Mean ± SD	27 ± 8.2		
Duration of employment (years)			
Median	1.3		
Mean ± SD	3.9 ± 5.3		
Estimated cumulative exposure (unit-days)^c^	22% worked < 90 days	43% worked 90 days to < 3 years	35% worked ≥3 years
Lowest tertile (0 to < 11,000)	753, 97%	634, 42%	105, 8%
Middle tertile	21, 3%	867, 57%	371, 30%
Highest tertile (≥90,000)	0	22, 1%	775, 62%
PYAR	108,930		

a Forty-one workers were missing employment dates, 1 stopped working before PCBs were used, 1 worked < 1 day, 26 were missing date of birth, and 5 were lost to follow-up before 1960.

b Subjects coded as “alive” include 104 persons with vital status unknown (considered alive until the date lost to follow-up).

c Estimated cumulative exposure could not be calculated for 21 workers with periods of unknown exposure level.

**Table 2:** Mortality in the NIOSH Indiana capacitor cohort for selected causes, by exposure tertile and overall, based on Indiana state rates for 1960–1998.

	Lowest tertile^a^			Middle tertile			Highest tertile			Overall b		
Underlying COD^c^ (ICD-9 codes)	*n*^d^	SMR	95% CI	*n*	SMR	95% CI	*n*	SMR	95% CI	*n*	SMR	95% CI
All cancers (140–208)	56	0.94	(0.7–1.2)	62	0.93	(0.7–1.2)	52	0.83	(0.6–1.1)	171	0.90	(0.8–1.0)
Buccal and pharyngeal (140–149)	2	1.97	(0.2–7.1)	0			1	0.88	(0.0–4.9)	3	0.90	(0.2–2.6)
Digestive system (150–159)	14	1.13	(0.6–1.9)	14	0.99	(0.5–1.7)	11	0.80	(0.4–1.4)	39	0.96	(0.7–1.3)
Esophagus (150)	2	1.47	(0.2–5.3)	2	1.27	(0.2–4.6)	3	1.95	(0.4–5.7)	7	1.55	(0.6–3.2)
Stomach (151)	3	2.40	(0.5–7.0)	0			2	1.47	(0.2–5.3)	5	1.23	(0.4–2.9)
Intestine (except rectum) (152–153)	7	1.43	(0.6–3.0)	4	0.72	(0.2–1.8)	4	0.74	(0.2–1.9)	15	0.94	(0.5–1.5)
Rectum (154)	0			1	0.97	(0.0–5.4)	0			1	0.34	(0.0–1.9)
Biliary passages, liver, and gallbladder (155, 156)	0			2	1.46	(0.2–5.3)	0			2	0.51	(0.1–1.8)
Pancreas (157)	2	0.77	(0.1–2.8)	5	1.69	(0.5–3.9)	2	0.70	(0.1–2.5)	9	1.06	(0.5–2.0)
Respiratory system (160–165)	16	0.77	(0.4–1.3)	24	0.98	(0.6–1.5)	19	0.79	(0.5–1.2)	59	0.85	(0.6–1.1)
Trachea, bronchus, and lung (162)	16	0.80	(0.5–1.3)	24	1.02	(0.7–1.5)	19	0.82	(0.5–1.3)	59	0.88	(0.7–1.1)
Breast (174–175)	4	1.04	(0.3–2.7)	3	0.92	(0.2–2.7)	0			8	0.83	(0.4–1.6)
Male genital organs (185–186)	1	0.43	(0.0–2.4)	2	0.69	(0.1–2.5)	1	0.32	(0.0–1.8)	4	0.47	(0.1–1.2)
Prostate (185)	1	0.48	(0.0–2.7)	2	0.76	(0.1–2.7)	1	0.33	(0.0–1.8)	4	0.51	(0.1–1.3)
Urinary organs (188–189)	1	0.39	(0.0–2.2)	1	0.34	(0.0–1.9)	2	0.71	(0.1–2.6)	4	0.48	(0.1–1.2)
Kidney (189.0–189.2)	0			1	0.55	(0.0–3.0)	1	0.59	(0.0–3.3)	2	0.38	(0.0–1.4)
Bladder and other urinary organs (188, 189.3–189.9)	1	1.11	(0.0–6.2)	0			1	0.87	(0.0–4.9)	2	0.63	(0.1–2.3)
Other and unspecified sites (170–173, 187, 190–199)	13	1.54	(0.8–2.6)	8	0.88	(0.4–1.7)	13	1.63	(0.9–2.8)	34	1.32	(0.9–1.9)
Melanoma (172)	5	3.72*	(1.2–8.7)	2	1.51	(0.2–5.4)	2	1.97	(0.2–7.1)	9	2.43*	(1.1–4.6)
Brain and nervous system (191–192)	3	1.38	(0.3–4.0)	4	1.79	(0.5–4.6)	5	2.71	(0.9–6.3)	12	1.91	(1.0–3.3)
Other and unspecified sites (187, 194–199)	5	1.25	(0.4–2.9)	1	0.22	(0.0–1.2)	5	1.17	(0.4–2.7)	11	0.86	(0.4–1.5)
Lymphatic and hematopoietic (200–208)	5	0.82	(0.3–1.9)	10	1.53	(0.7–2.8)	5	0.87	(0.3–2.0)	20	1.08	(0.7–1.7)
Non-Hodgkin lymphoma (200, 202)	1	0.42	(0.0–2.3)	5	1.93	(0.6–4.5)	3	1.30	(0.3–3.8)	9	1.23	(0.6–2.3)
Diabetes mellitus (250)	2	0.42	(0.1–1.5)	3	0.58	(0.1–1.7)	5	1.03	(0.3–2.4)	10	0.67	(0.3–1.2)
Blood and blood-forming diseases (281–289)	0			1	1.40	(0.0–7.8)	0			1	0.49	(0.0–2.7)
Alcoholism and mental disorders (290–319)	1	0.54	(0.0–3.0)	2	1.03	(0.1–3.7)	0			3	0.54	(0.1–1.6)
Nervous system diseases (320–337, 340–389)	0			3	0.82	(0.2–2.4)	2	0.60	(0.1–2.2)	5	0.47	(0.2–1.1)
Diseases of the heart (390–398, 402, 404, 410–414, 420–429)	48	0.76	(0.6–1.0)	64	0.85	(0.7–1.1)	67	0.90	(0.7–1.1)	179	0.83*	(0.7–1.0)
Ischemic heart disease (410–414, 429.2)	37	0.72*	(0.5–1.0)	58	0.94	(0.7–1.2)	54	0.88	(0.7–1.1)	149	0.84*	(0.7–1.0)
Cardiomyopathy (425)	7	2.79*	(1.1–5.7)	3	1.10	(0.2–3.2)	3	1.20	(0.2–3.5)	13	1.67	(0.9–2.9)
Other circulatory system (401, 403, 405, 415–417, 430–438, 440–459)	10	0.70	(0.3–1.3)	8	0.48*	(0.2–0.9)	8	0.48*	(0.2–1.0)	27	0.56**	(0.4–0.8)
Respiratory system (460–466, 470–478, 480–487, 490–519)	10	0.78	(0.4–1.4)	14	0.93	(0.5–1.6)	12	0.77	(0.4–1.3)	37	0.85	(0.6–1.2)
Digestive system (520–537, 540–543, 550–553, 555–558, 560, 562–579)	8	0.90	(0.4–1.8)	3	0.31*	(0.1–0.9)	3	0.35	(0.1–1.0)	14	0.51**	(0.3–0.9)
Cirrhosis of liver (571)	5	1.10	(0.4–2.6)	0			1	0.23	(0.0–1.3)	6	0.43*	(0.2–0.9)
Genitourinary system (580–608, 610, 611, 614–629)	2	0.80	(0.1–2.9)	0			1	0.37	(0.0–2.1)	3	0.37	(0.1–1.1)
Skin and subcutaneous tissue (680–686, 690–709)	0			1	5.94	(0.2–33.1)	0			1	2.09	(0.1–11.6)
Symptoms and ill-defined conditions (780–796, 798, 799)	2	1.24	(0.2–4.5)	1	0.64	(0.0–3.6)	0			3	0.69	(0.1–2.0)
Accidents (E800–E848, E850–E888, E890–E949)	19	0.85	(0.5–1.3)	12	0.59	(0.3–1.0)	14	1.18	(0.6–2.0)	45	0.82	(0.6–1.1)
Suicide (E950–E959)	6	0.72	(0.3–1.6)	6	0.77	(0.3–1.7)	7	1.41	(0.6–2.9)	19	0.90	(0.5–1.4)
Homicide (E960–E978)	2	0.71	(0.1–2.6)	1	0.42	(0.0–2.3)	0			3	0.46	(0.1–1.3)
HIV related (042–044)	3	3.16	(0.7–9.2)	0			0			3	1.42	(0.3–4.1)
Other causes (residual codes)	4	0.77	(0.2–2.0)	3	0.59	(0.1–1.7)	6	1.47	(0.5–3.2)	13	0.90	(0.5–1.5)
CODs not obtained	7			1			2			10		
All causes	180	0.84*	(0.7–1.0)	185	0.78**	(0.7–0.9)	179	0.83*	(0.7–1.0)	547^c^	0.81**	(0.7–0.9)

a Lowest tertile defined by cumulative exposure < 11,000 unit-days and highest tertile by cumulative exposure ≥ 90,000 unit-days.

b Total is greater than sum of tertiles because cumulative exposure could not be estimated for 21 workers, including 3 deceased workers with periods of employment lacking exposure data.

c Categories omitted because no deaths occurred include female genital organ cancers (ICD-9 codes 179–184), benign neoplasms (210–239), tuberculosis (010–018), and musculoskeletal diseases (710–721, 730).

d Observed number of deaths.

* *p* < 0.05.

** *p* < 0.01.

**Table 3:** NIOSH Indiana capacitor cohort: mortality from selected CODs according to estimated cumulative exposure to PCBs.

		Cumulative exposure (unit-days)						
		< 11,000		11,000–89,999		≥90,000		
Underlying COD		*n*	Ratio (95% CI)	*n*	Ratio (95% CI)	*n*	Ratio (95% CI)	Trend *p*-value
All causes	SMR^a^	180	0.84 (0.7–1.0)*	185	0.78 (0.7–0.9)**	179	0.83 (0.7–1.0)*	0.84
All cancers	SMR	56	0.94 (0.7–1.2)	62	0.93 (0.7–1.2)	52	0.83 (0.6–1.1)	0.48
Melanoma	SMR	5	3.72 (1.2–8.7)*	2	1.51 (0.2–5.4)	2	1.97 (0.2–7.1)	0.62
	SRR		1		0.38 (0.1–2.0)		0.58 (0.1–3.5)	0.72
	RR^b^		1		0.43 (0.1–2.3)		0.59 (0.1–3.2)	0.71
Brain cancer	SMR	3	1.38 (0.3–4.1)	4	1.79 (0.5–4.6)	5	2.71 (0.9–6.3)	0.34
	SRR		1		1.01 (0.2–4.6)		1.48 (0.3–6.4)	0.016
	RR		1		1.29 (0.3–5.8)		1.95 (0.4–8.5)	0.37

a SMR using Indiana rates.

b Obtained via Poisson regression, adjusted for sex, age (< 50, ≥50 years), calendar year (before 1980, after 1980), and latency (< 10, 10–19, ≥20 years).

* *p* < 0.05.

** *p* < 0.01.
