# Supplementary material for: Mortality among Workers Exposed to Polychlorinated Biphenyls (PCBs) in an Electrical Capacitor Manufacturing Plant in Indiana: An Update
Source: Environ Health Perspect. 2005 Sep 1;114(1):18–23. doi: 10.1289/ehp.8253 (PMC1332650; doi:10.1289/ehp.8253)
Supplement: Supplementary Material [file ehp0114-000018s1.pdf]

Mortality Studies Among Capacitor and Transformer Manufacturing Workers Exposed to PCBs, Grouped by Study Site

Site	Reference	N	Study design	Eligibility criteria	Study dates	PCB Exposure (PCB type if stated)	Other exposure	Cohort deaths, ratio[a] (CI)	Hi exp =	Hi exp deaths, ratio† (CI)
Canada, Ontario, transformer manufacture and repair	(Liss 1989)	1073: 273 women 800 men	cohort	≥ 1 year work 1960-1976	-1985	1978 air samples 3-8 μg/m^3	Benzene ca 45, SMR 1.2 (0.9-1.6)	146, SMR 1.0 (0.8-1.1);	Men ever in high exp job	24, SMR 0.8 (0.5-1.2); cancer 6, SMR 0.8 (0.3-1.9), lymph & hemat cancer 2 SMR 2.9 (0.3-12.4)
Canada, transformer manufacture	(Yassi et al. 1994)	2222: all men	cohort	≥ 1 month work 1946-1975	-1989	>27,000 gallons Askarels used in mfg	mineral oil, solvents	63-171[b], SMR 0.7-1.1 (nss); cancer 18-36, SMR 0.8-1.2 (nss);GI ca 6-17, SMR 0. 9-1.6 (nss); pancreas ca 5-11 SMR 2.9-7.6 (all ss)	Transformer assembly	35, SMR 0.9 (0.6-1.2); cancer 11, SMR 1.4 (0.7-2.5); GI cancer 5, SMR 2.3 (0.7-5.3); pancreas cancer 4, SMR 9.8 (2.6-25)
Italy, Milan vicinity, capacitor manufacture	(Bertazzi et al. 1982)	1310: 1020 women, 290 men	cohort	≥ 6 months work 1946-1978 production dept.	1978	1954 air samples 5200-6800 μg/m^3, 1977 TWA air samples 48-275 μg/m^3, surface wipes 0.2-159 μg/cm^2, dermal wipes 0.3-9.2 μg/cm^2, blood levels (n=67) 230.5± 174.5 ppb 54% chlorine PCBs; 114.1 ±79.6 ppb 42% chlorine PCBs; use stopped 1979		27, SMR 1.3 (0.9-1.9), ca 14, SMR 2.5 (1.4-4.2); lymph-hemat 4, SMR 4.4 (1.2-12.2)		
	(Bertazzi et al. 1987)	2100: 1556 women, 544 men	cohort	≥ 1 week work 1946-1978	1982	1982 surface wipes 0.003-6.3 μg/cm^2, dermal wipes 0.09-1.5 μg/cm^2, blood levels (n=37) 202.8±111.7 ppb 54% chlorine PCBs; 42.9 ±34.7 ppb 42% chlorine PCBs	TCE	64, SMR 1.2 (0.9-1.5), cancer 26, SMR 2.0 (1.3-2.9); lymph-hemat 7, SMR 3.0 (1.2-6.5); GI tract (men) 6, SMR 3.5 (1.3-8.1)		
	(Tironi et al. 1996)	1556: all women	cohort	≥ 1 week work 1946-1980, production dept.	1954-1982	1954 air samples 5200-6800 μg/m^3 , 1977 TWA air samples 48-275 μg/m^3, surface wipes 0.2-159 μg/cm^2, dermal wipes 0.3-9.2 μg/cm^2, blood levels (n=67) 230.5± 174.5 ppb 54% chlorine PCBs; 114.1 ±79.6 ppb 42% chlorine PCBs; use stopped 1980		47, SMR 1.4 (1.0-1.8), cancer 19, SMR 1.2 (0.7-1.8); lymph-hemat 5, SMR 1.4 (0.5-3.3)		
Sweden, capacitor manufacture	(Gustavsson et al. 1986)	142: all men	cohort	≥ 6 mo. work 1965-1978, Swedish	1982	42% chlorination		21, RR 1.0 (0.6-1.5); cancer 7, RR 1.3 (0.5-2.7)	Capacitor filler, repairer	"no tendency toward increased mortality"
	(Gustavsson and Hogstedt 1997)	242: all men	cohort	≥ 6 mo. work 1965-1978	1965-1991	Tasks IDd as low, med, hi PCB		56, SMR 1.2 (0.9-1.5); cancer 16, SMR 1.3 (0.8-2.2)	Impregnation & repair	23, SMR 1.6 (1.0-2.3); cancer 7, SMR 1.9 (0.8-3.9)
USA, Illinois, capacitor manufacture	(Mallin et al. 2004)	2885: 1707 women, 1178 men	cohort	At least 1 day in ≥ 1 quarter 1944-1977	1944-2000	Arochlor 1254 1952-1956 Arochlor 1242 1954-1970 Arochlor 1016 1970-1979	Chlorinated naphthalenes 1944-1981, di-2-ethylhexyl phthal ate, 1979-1981	1546, SMR 1.01 (1.0-1.1); ca 347, SMR 1.10 (1.0-1.2); liver, biliary 14, SMR 2.38 (1.3-4.1); stomach 17, SMR 1.89 (1.1-3.1)	>5 yrs between 1952-1977	Male stomach 3, SMR 3.09 (0.6-9.0); female intestine 9, 2.25 (1.0-4.3); female liver 4, SMR 5.57 (1.5-14.3)

Site	Reference	N	Study design	Eligibility criteria	Study dates	PCB Exposure (PCB type if stated)	Other exposure	Cohort deaths, ratio[a] (CI)	Hi exp =	Hi exp deaths, ratio† (CI)
USA, Indiana, capacitor manufacture	(Sinks et al. 1992)	3588: 846 women, 2742 men	cohort	≥ 1 day work 1957-1977, known or presumed Caucasian, work history complete	1957-1986	1977 air samples: zone 5 personal 94±68 μg/m^3 , area 76±52 μg/m^3 ; zone 3 area 59±19 μg/m^3 ; zone 2 area 48±13 μg/m^3 ; zone 1 area 16±15 μg/m^3 ; 1977 serum: only zone 5, 763± 1117 ng/ml; only zone 3, 100±27 ng/ml; only zone 2, 121±61 ng/ml	1,1,1-trichloro-ethane, TCE, MEK, toluene, xylene	192, SMR 0.7 (0.6-0.8); cancer 54, SMR 0.8 (0.6-1.1); skin 8, SMR 4.1 (1.8-8.0); brain 5, SMR 1.8 (0.6-4.2)	≥10 yrs work	Skin cancer 3, SMR 7.0 (1.4-23); brain cancer 3, SMR 4.8 (1.0-16)
USA, Massachusetts, New Bedford, capacitor manufacture	(Brown & Jones 1981)	1599: 924 women 675 men	cohort	≥ 3 mo. work 1946-1976, possible PCB exposure, no TCE exposure	-1976	1977 TWA personal air samples 170-1260 μg/m^3 , area samples 50-810 μg/m^3	TCE exposure (at degreasers, 53-78 ppm)	90, SMR 0.9 (0.7-1.1); cancer 26, SMR 1.0 (0.6-1.4); rectal 3, SMR 4.3 (0.9-14); liver 2, SMR 3.0 (0.3-13); cirrhosis 5, SMR 1.6 (0.5-3.9)	entire cohort	
	(Brown 1987)	1607: 930 women, 677 men	cohort	≥ 3 mo. work 1946-1977, possible PCB exposure, no TCE exposure	-1982	1977 TWA personal air samples 170-1260 μg/m^3, area samples 50-810 μg/m^3	Degreaser TCE exposure, 53-78 ppm	179, SMR 1.0 (0.8-1.1); cancer 44, SMR 0.9 (0.7-1.2); rectal 3, SMR 2.7 (0.6-1.2); liver 4, SMR 3.3 (0.9-9.3)	entire cohort	Excess liver ca in women: 4, SMR 4.4 (1.2-12.3)
USA, New York, capacitor manufacture	(Brown & Jones 1981)	968: 385 women, 583 men	cohort	≥ 3 mo. work 1938-1976, possible PCB exposure, no TCE exposure	-1976	1977 TWA personal air samples 24-393 μg/m^3 , area samples 3-476 μg/m^3	Degreaser TCE exposure, <35 - 321 ppm	73, SMR 1.0 (0.8-1.2); cancer 13, SMR 0.8 (0.4-1.3); rectal 1, SMR 2.1 (0.0-17); liver 1, SMR 2.4 (0.0-20); cirrhosis 1, SMR 0.4 (0.0-3.4)	entire cohort	
	(Brown 1987)	981: 383 women, 593 men	cohort	≥ 3 mo. work 1938-1977, possible PCB exposure, no TCE exposure	-1982	1977 TWA personal air samples 24-393 μg/m^3 , area samples 3-476 μg/m^3	Degreaser TCE exposure, <35 - 321 ppm	116, SMR 0.9 (0.7-1.1); cancer 18, SMR 0.6 (0.3-0.9); rectal 1, SMR 1.2 (0.0-10); liver 1, SMR 0.7 (0.0-5.4)	entire cohort	
USA, Massachusetts, Pittsfield, transformer manufacture	(Greenland et al. 1994)	~3500 ever employed	death certificate study	Worked 1930s-1984, vested in pension fund, white male, job history available	1969-1984	Pyranol transformer oil	Trichlorobenzene, benzene, TCE, other solvents, machining fluids, asbestos, resin systems	1821 all cause, 513 cancer, 33 pancreas cancer, 37 lymph-hematopoietic	Any vs. none	Liver-biliary OR 2.4 (0.6-9.7),lymphomas OR 3.3 (1.1-9.3) for PCB exposure

Site	Reference	N	Study design	Eligibility criteria	Study dates	PCB Exposure (PCB type if stated)	Other exposure	Cohort deaths, ratio[a] (CI)	Hi exp =	Hi exp deaths, ratio† (CI)
USA, New York, capacitor manufacture	(Brown & Jones 1981)	968: 385 women, 583 men	cohort	≥ 3 mo. work 1938-1976, possible PCB exposure, no TCE exposure	-1976	1977 TWA personal air samples 24-393 μg/m^3, area samples 3-476 μg/m^3	Degreaser TCE exposure, <35 - 321 ppm	73, SMR 1.0 (0.8-1.2); cancer 13, SMR 0.8 (0.4-1.3); rectal 1, SMR 2.1 (0.0-17); liver 1, SMR 2.4 (0.0-20); cirrhosis 1, SMR 0.4 (0.0-3.4)	entire cohort	
	(Brown 1987)	981: 383 women, 593 men	cohort	≥ 3 mo. work 1938-1977, possible PCB exposure, no TCE exposure	-1982	1977 TWA personal air samples 24-393 μg/m^3, area samples 3-476 μg/m^3	Degreaser TCE exposure, <35 - 321 ppm	116, SMR 0.9 (0.7-1.1); cancer 18, SMR 0.6 (0.3-0.9); rectal 1, SMR 1.2 (0.0-10); liver 1, SMR 0.7 (0.0-5.4)	entire cohort	
	(Taylor et al. 1988)	6292: 2691 women, 3601 men	cohort	≥ 3 months work 1946-1975	-1980	1975 air samples geometric mean (GM) 679 μg/m^3 direct exposure areas; GM 260 μg/m^3 indirect exposure areas; 1977 air samples GM 310 μg/m^3 direct exposure areas; GM 27 μg/m^3 indirect exposure areas; 1977 personal air samples GM 168 μg/m^3 direct exposure areas		510, SMR 0.83 (0.76, 0.91); cancer 136, SMR 0.9 (0.8-1.1); GI cancer 44, SMR 1.3 (1.0-1.8); skin 3, SMR 1.0 (0.2-3.2); brain 1, SMR 0.2 (0.0-1.6); lymph-hemat 10, SMR 0.7 (0.3-1.3)		
	(Kimbrough et al. 1999)	7075: 3013 women, 4062 men	cohort	≥ 3 months work 1946-1977	1946-1993	Hi exp: Filling & impregnating 1975 air samples 227-1500 μg/m^3		1195, SMR 0.8 (0.7-0.8); cancer 353, SMR 0.9 (0.8-1.0); rectal 10, SMR 1.3 (0.6-2.5); liver 5, SMR 0.9 (0.3-2.0)	Hourly workers	966, SMR 0.9(0.8-0.9); ca 278, SMR 1.0 (0.8-1.1); rectal 7, SMR 1.2 (0.5-2.6); liver 4, SMR 0.9 (0.2-2.4)
	(Kimbrough et al. 2003)	7075: 3013 women, 4062 men	cohort	≥ 3 months work 1946-1977	1946-1998	Hi exp: Filling & impregnating 1975 air samples 227-1500 μg/m^3		1654, SMR 0.9 (0.85-0.93); cancer 492 SMR 1.0 (0.9-1.1); rectal 12, SMR 1.3 (0.7-2.3); liver 9, SMR 0.8 (0.4-1.5)	Hourly workers	1333, SMR 1.0 (0.9-1.0); ca 381, SMR 1.0 (0.9-1.2); rectal 8, SMR 1.2 (0.5-2.4); liver 8, SMR 1.0 (0.4-1.9)

a. Odds ratio (OR), standardized mortality ratio (SMR), or relative risk (RR).
b. Yassi used various eligibility criteria and calculated SMRs for each set.

References

Bertazzi PA, Riboldi L, Pesatori A, Radice L, Zocchetti C. 1987. Cancer mortality of capacitor manufacturing workers. Am J Ind Med 11: 165-76.

Bertazzi PA, Zocchetti C, Guercilena S, Della Foglia M, Pesatori AC,Riboldi L (1982) Mortality study of male and female workers exposed to PCBs. In: Prevention of Occupational Cancer--International Symposium pp. 242-248. International Labour Office, Geneva.

Brown DP. 1987. Mortality of workers exposed to polychlorinated biphenyls--an update. Arch Environ Health 42: 333-9.

Brown DP, Jones M. 1981. Mortality and industrial hygiene study of workers exposed to polychlorinated biphenyls. Arch Environ Health 36: 120-9.

Greenland S, Salvan A, Wegman DH, Hallock MF, Smith TJ. 1994. A case-control study of cancer mortality at a transformer-assembly facility. Int Arch Occup Environ Health 66: 49-54.

Gustavsson P, Hogstedt C. 1997. A cohort study of Swedish capacitor manufacturing workers exposed to polychlorinated biphenyls (PCBs). Am J Ind Med 32: 234-9.

Gustavsson P, Hogstedt C, Rappe C. 1986. Short-term mortality and cancer incidence in capacitor manufacturing workers exposed to polychlorinated biphenyls (PCBs). Am J Ind Med 10: 341-4.

Kimbrough RD, Doemland ML, LeVois ME. 1999. Mortality in male and female capacitor workers exposed to polychlorinated biphenyls. J Occup Environ Med 41: 161-71.

Kimbrough RD, Doemland ML, Mandel JS. 2003. A mortality update of male and female capacitor workers exposed to polychlorinated biphenyls. J Occup Environ Med 45: 271-82.

Liss GM. 1989. Mortality and Cancer Morbidity Among Transformer Manufacturing Workers . Toronto, Canada: Ontario Ministry of Labour Policy and Regulations Branch Health Studies Service.

Mallin K, McCann K, D'Aloisio A, Freels S, Piorkowski J, Dimos J et al. 2004. Cohort mortality study of capacitor manufacturing workers, 1944-2000. J Occup Environ Med 46: 565-76.

Sinks T, Steele G, Smith AB, Watkins K, Shults RA. 1992. Mortality among workers exposed to polychlorinated biphenyls. Am J Epidemiol 136: 389-98.

Taylor PR, Stelma JM, Auger I, Lawrence CE. 1988. The relation of occupational polychlorinated biphenyl exposure to cancer and total mortality. Cambridge, Massachusetts: Harvard School of Public Health.

Tironi A, Pesatori A, Consonni D, Zocchetti C, Bertazzi PA. 1996. Mortalita di lavoratrici esposte a PCB [The mortality of female workers exposed to PCBs]. Epidemiol Prev 20: 200-2.

Yassi A, Tate R, Fish D. 1994. Cancer mortality in workers employed at a transformer manufacturing plant. Am J Ind Med 25: 425-37.
